# Comparing apples and pears: misleading conclusions about the population mental health impact of a parenting programme, a commentary on Marryat, Thompson and Wilson (2017)

**DOI:** 10.1186/s12887-019-1570-z

**Published:** 2019-08-05

**Authors:** Matthew R. Sanders, Linda de Caestecker, Stephen McLeod, Jamin J. Day, Karen M. T. Turner, Alina Morawska, James Kirby

**Affiliations:** 1 Parenting and Family Support Centre, the University of Queensland, Brisbane, Australia; 2 National Health Service Greater Glasgow and Clyde, Glasgow, Scotland

**Keywords:** Child mental health, Population impact, Parenting, Triple P

## Abstract

**Background:**

The article by Marryat, Thompson and Wilson (2017) in *BMC Pediatrics* presents an evaluation of the implementation of the Triple P system as a public health intervention conducted by the Glasgow City Council and NHS Greater Glasgow and Clyde.

**Discussion:**

Unfortunately, the conclusions drawn are questionable for multiple reasons. The lack of a controlled design precludes defensible conclusions about intervention effects free from routine threats to internal validity. There was a substantial mismatch between the intervention sample and the population sample assessed. The article’s title and abstract leave readers with the mistaken impression that the children assessed for outcome were suitably representative of intervention families, when in fact many of the children in the intervention families were missing from the teacher-report outcome assessment (a single questionnaire), and many or most of the children in the teacher-report outcome assessment belonged to families who had never received the intervention. Although Triple P targets parent-child relations and child behavioural and emotional problems at home, Marryat et al. narrowly defined mental health impact as child difficulties in nursery or preschool, while not reporting data from practitioners and parents in the same evaluation that did not support the authors’ conclusion. The paper was further diminished by a number of misleading statements and factual errors related for example to other research on Triple P.

**Summary:**

Studying the extent to which child mental health functioning at home can generalise to school settings is an important topic of inquiry in relation to parenting support interventions, but unfortunately the Marryat et al. article did not move this area forward.

## Background

The Triple P — Positive Parenting Program (Triple P) [1] is a multilevel system of parenting support designed to prevent and treat child social, emotional and behavioural problems. The Triple P system involves a population health approach to parenting support, and in recent years has been evaluated at a population level in various locations including the United States [2], Australia [3], and Ireland [4]. The population approach involves universal access to evidence-based parenting support through a mix of prevention, early intervention and targeted intervention options, with the aim of providing parents with the minimal amount of support they need.

A recent paper published in *BMC Pediatrics* [5] reports on a city-wide implementation of Triple P in Glasgow, Scotland. We applaud the authors for conducting an independent evaluation of the Triple P system, and endorse the value of independent evaluations of parenting support interventions. A healthy mix of independent and developer-led evaluations is vital for the ongoing refinement and dissemination of rigorous, evidence-based practice in the field of parenting support interventions. We also welcome the focus on generalisation of the effects of parenting programs to other contexts, as this has important implications for the range of services that are offered to communities. However, following a careful review of the methodology and findings reflected in this paper, we have concerns relating to the validity of inferences drawn by the authors, namely that “no convincing evidence of benefit for preschool aged children’s mental health problems” was found from the initiative. We believe this conclusion is untenable due to the methodological, conceptual, and measurement limitations of the uncontrolled study, which we detail below. There are also numerous misleading claims and factual errors throughout, which further undermine the paper’s validity.

## Main text

### Uncontrolled design

The Marryat et al. [5] design neglected to include a viable control condition. The lack of a control or comparison group precludes any conclusions about program effects that are free from routine threats to internal validity. Although briefly mentioning this as a weakness, the authors maintain that prior survey data make a control condition unnecessary, and support this notion by pointing out sampling and other design challenges encountered in prior experimental studies. Challenges encountered in prior controlled studies do not mitigate the absence of a control group in the Marryat et al. (2017) study. This methodological limitation is further compounded by the fact that delivery of Triple P had already started in the target city prior to the launch of this study, further suggesting the importance of including a no-intervention control condition. The authors claimed that it is highly unlikely the prior delivery of Triple P affected baseline data, but they provided no evidence for this assertion.

### Mismatch between intervention and outcome measurement age

One of the most serious methodological problems with the article has to do with the age range of the children assessed. The article fails to make it clear to the reader that there is a substantial mismatch between the intervention and the outcome measurement with respect to child age range and sampling. Parental participation in the intervention (i.e., the independent variable) targeted children 2-16 years of age, while the teacher-reported outcome variable (i.e., the dependent variable) assessed 4 and 5 year-olds. The article provided detail about the number of families participating in the intervention, and briefly mentioned that a substantial proportion (i.e., 40% or more) of the families that received parenting services had children that were too old to be picked up by the outcome measure. The 40% figure refers to the percentage of children who were older than age five at the time their parents participated in the intervention. However, the implications of attempting to detect population-level impact by assessing a diluted, marginal sample of those who actually received the intervention have not been discussed.

Related to the age and sample mismatch issue, many of the families receiving the parenting intervention did not have a child within an eligible age-range for inclusion in the teacher-report outcome assessment, and thus were not represented in the evaluation of the intervention. Likewise, many of the children assessed via teacher report were from families who had never received the intervention. No data were provided regarding the proportion of children assessed that had a parent who had participated in Triple P. This omission, along with the aforementioned lack of control condition, makes it impossible to calculate common indices representative of population-level impact, such as risk ratios or number-needed-to-treat.

### Narrow focus for assessing mental health impact

Triple P aims to reduce child behavioural and emotional difficulties through the mechanism of promoting change in parenting practices, and thus the primary focus is on producing change in the family context. Although Marryat et al. [5] claimed to evaluate the mental health impact of Triple P, they presented data related only to child difficulties at school (in this case, the nursery or pre-school context) via a routinely collected teacher-report questionnaire, the Strengths and Difficulties Questionnaire (SDQ) [6]. This narrow focus is important because (a) changes within the school setting are not the primary target of the Triple P intervention, and (b) the aims and conclusions outlined by the authors do not align with the actual data reported.

The impact of family-based interventions like Triple P on school adjustment is an important research question, and one that would be reasonable to explore. We might anticipate that significant improvements in child mental health or behavioural difficulties seen within the home context might also be seen at school, particularly if the child has significant difficulties at school in the first place. However, reliance on teacher-report data as the sole indicator of population-level impact on child mental health is seriously flawed. First, there are generally low levels of concordance between teacher and parent reports regarding child difficulties, with often only modest correlation (e.g., < .30) between parents and teachers as informants, and teachers typically reporting fewer problems overall (e.g. [7, 8]).

Teacher report cannot be used as a proxy for parents’ experiences with their children at home or for parental reports on children’s mental health status. To support the decision to present only teacher data, the authors claimed that reliance on parental report can be problematic due to its potential to introduce a measurement confound with parent’s mental state, however no evidence is presented that teacher-reported data provides a more realistic or reliable indication of children’s mental health or difficult behaviours than parent-reported data. Teacher-report data can provide a valuable contribution within a multi-informant approach to understanding the broader impact of a parenting intervention such as Triple P, yet as with any single-informant approach to data collection, findings should be framed within the confines of the extent to which these are generalisable—in this case, teachers’ views of child behaviour within the preschool setting posited to generalise to the home, and children’s general mental health. We acknowledge that issues of pragmatism can preclude the collection of data from multiple sources, but the authors failed to acknowledge this limitation. The result, unfortunately, took the form of over-reaching and inappropriately generalised conclusions regarding the population-level impact on mental health.

### Selective reporting

Original data from the final report of the Glasgow Parenting Support Framework Evaluation [9] included parent-reported outcomes for pre-school children using the SDQ. Although not aggregate-level data, these data showed positive outcomes for Triple P when parents completed the program. However, these findings and other qualitative data from practitioners and parents have been ignored, which is problematic because reporting the full pattern of findings would have given the reader a more complete understanding that perhaps would have contradicted the authors’ stated conclusions. Furthermore, the authors claimed there were no changes in social outcomes for children, but they only examined the Conduct Problems subscale when analysing the SDQ data, and not other SDQ subscales. The authors directed the reader to Additional File 4: Table S1 for further information regarding the pattern of differences in subscales other than the Total Difficulties score and Conduct Problems subscale, yet this table includes mean differences on only SDQ Total score and no individual subscale information. Subscales are plotted individually in Additional File 3: Fig. S1, however the lack of accompanying statistical information hinders any substantive interpretation of the data.

### Factual errors and misleading statements

The authors’ claim that independent observers do not generally report positive findings is incorrect. Sanders, Kirby, Tellegen, and Day [10], who conducted the most comprehensive meta-analysis of 101 studies on Triple P, found significant intervention effects across 21 studies reporting observational data on child behavior, including both prevention and treatment studies, with an average effect size (Cohen’s d) of .50. Similar positive effects on observational measures of child behavior were found in a more recent meta-analysis of Stepping Stones Triple P by Ruanne and Carr [11], who reported an average effect size of .51. The reported effect sizes for independent observational measures do not align with Marryat, Thompson and Wilsons’s claim of no impact.

The authors claimed that Triple P has little effect in deprived communities. This claim ignores studies showing that socioeconomic status does not moderate effect sizes for child outcomes in Triple P studies [10] and the mounting evidence that Triple P works well in low resource communities (e.g. [2, 4, 12]). There have since been a number of high quality studies showing the value of Triple P in a range of disadvantaged communities. Examples include: a place-based randomised trial of the Triple P system in the US showing population level effects on child maltreatment in communities with substantial representation of disadvantaged families [2]; an RCT of low intensity Triple P Discussion Groups in Panama showing positive effects on child and parent outcomes with parents in deprived communities [12]; an RCT of Triple P Discussion Groups with a Maori indigenous population in New Zealand [13]; evaluations of Group Triple P with Aboriginal and Torres Strait Islander samples in Australia [14]; and a trial of Triple P Online with vulnerable disadvantaged urban mainly African American and Latino families in Los Angeles [15]. Qualitative studies showing high levels of consumer acceptance of Triple P principles and techniques have been conducted with homeless parents [16], vulnerable low income families involved with child protective services [15], and women in shelters who have histories of domestic violence [17]. Fives et al. [4] reported that many participants in the Ireland population roll out of Triple P were low SES (39% of Group Triple P participants, 33% of workshop participants, and 26% of seminar participants had a medical card, a key indicator of low SES). Contrary to Marryat et al.’s conclusion [5], Triple P has been found to be a promising intervention with many vulnerable, socially disadvantaged parents.

The paper also raised concerns about the costs of Triple P without defining the costs or placing the costs in perspective relative to not intervening, or the costs of other intervention strategies. Serving 10,000 families ostensibly costs more than serving 100 families, but the key metric would be the per-family cost, which the article ignored in making a general pronouncement (i.e., “consumes substantial resources”). It did not discuss the potential cost savings of brief, early, minimal intervention, or the mix of varied delivery formats, for example, the cost saving in offering group programs serving several families in the same amount of staff time as conducting individual sessions.

The paper failed to take into account that during the intervention period in the same catchment area other parenting interventions were also being supported and implemented concurrently, albeit on a smaller scale. This again highlights the need for control data to allow suitable comparisons to support conclusions around population-level impact of any universal prevention or public health initiative.

Finally, there are some major errors in the article. Firstly, it reports null results of “a recent cluster randomized control trial exploring the impact of Triple P levels 2 and 3 on pre-schoolers’ externalizing behaviours and parental mental health”. The references cited relate to Hiscock et al. [18], a study that was not a Triple P intervention, and Malti et al. [19], a study that tested one level of Triple P (Level 4 Group). Similarly, the article refers to Prinz and Sanders [20] in relation to “previous work in which no significant improvement in child-based outcomes resulted from a public health parenting programme” which is an incorrect citation—the article cited is a theoretical piece about population-level interventions and does not include an evaluation nor any discussion of child outcome results. The authors also cite a study reporting a subgroup analysis focusing on lone parent families that showed no benefit from the Triple P intervention [21]. It is true the study reported no group difference between intervention and control parents around parenting and child behaviour based on self-report data. However, independent clinical observations reported within the same paper showed significant improvements in positive parenting behaviour and decreases in negative child behaviour for the intervention group. We find it curious that this finding was omitted from the authors’ discussion, particularly considering it reports data from an independent source which would seem of relevance given the prior arguments made by the authors.

The paper claimed to have registered the study protocol, yet the reference list only cites a University of Glasgow webpage for a description of the protocol, no trial registration number. Furthermore, the protocol as described is significantly different from the primary findings reported in the paper or the final evaluation report.

### Measurement problems

The study had a number of measurement problems. First, one of the primary outcome measures was a modified version of the Conduct Problems subscale of the SDQ. Using only three of the original five items for this scale resulted in a modified version that had low internal consistency (α = 0.66), which is below the commonly accepted threshold (0.7) for scientific acceptability, and which relied on a questionably small number of items (three). Additionally, they use a weighted procedure to compute an average score for this modified subscale, and then applied the standard cut-off levels intended for the full subscale. Given these measurement issues the validity of this scale as a primary outcome variable is uncertain and highly questionable.

## Conclusions

Overall, while an independent evaluation of a complex community-wide intervention such as that undertaken in Glasgow is welcome, the capacity to learn from the present evaluation is diminished by methodological, interpretational and factual issues and errors. Given the absence of a proper control or comparison group, and in light of the substantial mismatch between the intervention sample and the outcome measurement age group, rather than sweeping claims of “No evidence of whole population mental health impact,” the scientifically justifiable conclusion is one of uncertainty. It is not possible to assert with any confidence that the observed data reflect a true test of intervention impact (i.e. behavior assessed in the preschool setting, with assumptions unable to be drawn about the home setting) or an inadequate or limited test of intervention impact (i.e. questionable measurement validity and a lack of suitable control condition). This article provides further support for the pressing need for the field to develop accurate and scalable measurement procedures to test population effects for public health interventions.

The ongoing delivery of Triple P in Glasgow is viewed by the NHS as part of a long-term strategy and it was expected to take several years for any new programme to be properly established in practice. In a city with Glasgow’s levels of poverty and deprivation, health visitors implementing the programme have spent time engaging parents and helping them understand the need and benefit of parenting support. As expected, there have been many learnings over the years since Triple P was first introduced, including the need for dedicated practitioners within health visiting teams to run parenting groups and to establish strong links and partnerships with the voluntary sector to further improve engagement. Although positive outcomes have been achieved with many individual families who completed the programme, sustained implementation of Triple P requires a quality improvement framework that has been adopted by the implementation team in Glasgow. This involves applying learnings from implementation science, large-scale rollouts of the Triple P system (e.g. [4, 22]), consumer and end user feedback from parents and practitioners, and outcome data collected as part of routine implementation. The ultimate aim is to continuously improve fidelity of delivery, minimise drop out and increase the reach and impact of the intervention.

## Abbreviations

NHS: National Health Service; SDQ: Strengths and Difficulties Questionnaire; Triple P: Triple P—Positive Parenting Program

## Acknowledgements

Not applicable.

## Authors’ contributions

All authors made substantial intellectual contributions to the conception, drafting, and critical revision of this manuscript, and have given final approval for its publication.

## Funding

Not applicable.

## Availability of data and materials

Data sharing is not applicable to this article as no datasets were generated or analysed during the current study.

## Ethics approval and consent to participate

Not applicable.

## Consent for publication

Not applicable.

## Competing interests

The Triple P – Positive Parenting Program is owned by The University of Queensland (UQ). The University through its main technology transfer company, UniQuest Pty Ltd., has licensed Triple P International Pty Ltd. to publish and disseminate the program worldwide. Royalties stemming from published Triple P resources are distributed to the Faculty of Health and Behavioural Sciences at UQ, Parenting and Family Support Centre, School of Psychology at UQ, and contributory authors. No author has any share or ownership in Triple P International Pty Ltd. MS, KT and AM are contributory authors of disseminated Triple P resources and receive royalties as specified above. MS, KT, JD and AM receive salary from the Parenting and Family Support Centre; MS is a consultant to Triple P International. LdC, SM, and JK have no financial competing interests to declare. MS, JD, KT, AM and JK are members of the Triple P Research Network (TPRN), a non-financial body formed to foster international collaborations and knowledge sharing between researchers interested in parent and family research including the Triple P—Positive Parenting Program.

## Availability of Data and Materials

Data sharing is not applicable to this article as no datasets were generated or analysed during the current study.
