# Publisher Correction to: Comparing apples and pears: misleading conclusions about the population mental health impact of a parenting programme, a commentary on Marryat, Thompson and Wilson (2017)

**DOI:** 10.1186/s12887-019-1667-4

**Published:** 2019-10-15

**Authors:** BMC Pediatrics

**Affiliations:** 0000 0004 0544 054X grid.431362.1 BMC, London, UK

**Publisher Correction to: BMC Pediatr**

**https://doi.org/10.1186/s12887-019-1570-z**

The original version of this article [1] was published in error before the text of the article had been finalised. The article has now been corrected. The publisher apologizes for the inconvenience caused to the authors and readers.
