# Supplementary material for: Comparing apples and pears: misleading conclusions about the population mental health impact of a parenting programme, a commentary on Marryat, Thompson and Wilson (2017)
Source: BMC Pediatr. 2019 Aug 5;19:269. doi: 10.1186/s12887-019-1570-z (PMC6681483; doi:10.1186/s12887-019-1570-z)
Supplement: Related texts

# Reply: Authors’ response

**Authors:** Louise Marryat, Lucy Thompson, Philip Wilson

In the above correspondence, Sanders et al. have commented on our paper [5] which reported a lack of impact of whole-population implementation Triple P in Glasgow City. Sanders et al. consider the findings not proven due to ‘methodological, interpretational and factual issues and errors’.

The main criticisms are:

1. The design neglected to include a control condition
2. The mismatch between the age of the target child for the intervention and the age of population assessment
3. The focus on the outcome as childhood mental health difficulties being too narrow
4. Selective reporting of outcomes
5. Factual errors and misleading statements
6. A number of measurement problems

We strongly reject the contention that our conclusions were not justified by the evidence. Whilst our research has weaknesses, as with any evaluation carried out in the real world, these were clearly set out in the original paper, and do not alter our overall conclusions. We now discuss each of the arguments in turn.

## Assertion 1 the design neglected to include a variable control condition

It is true that the study design did not have a control group. The authors acknowledged this as a potential weakness in the original manuscript. The resources available to us, administered through NHS Greater Glasgow and Clyde (NHS GGC), were insufficient to meet the cost of a control group, and data collection had not begun at the start of the intervention, so no pre-intervention comparison group was available.

The study design was subject to a review process. The evaluation steering group, which included NHS members, sent the protocol out to external peer review by Warwick Medical School, who provided very strong support for our design.

The conclusions reached in the Glasgow evaluation are supported by the only other published independent UK evaluation of Triple P, a randomised controlled trial showing no effect from Triple P interventions [23].

## Assertion 2 the mismatch between the age of the target child for the intervention and the age of population assessment

This criticism refers to the fact that the six years’ of population outcome measures of child mental health difficulties were assessed at age 4-5 years, whereas the group Triple P interventions were delivered to parents of children of a range of ages, many of whom were over that age. This argument has little relevance to our conclusions. First, group Triple P was only one part of a *population-level* programme. Triple P International, in its tender documents shared with NHS GGC in 2010, considered the level of reach of the programme was sufficient to produce a whole-population effect and that effect should have been seen at any and all ages of children. Second, the Glasgow Triple P programme included a city-wide media campaign involving television, newspaper and billboard posters aimed at all parents as well as a universal seminar programme. It is difficult to believe that any Glasgow family was not exposed to at least some of these Triple P materials. Third, parents nominated only one of their children as the index child when attending groups, and it was this child for whom the age was recorded in our process evaluation. Many attending parents would have had younger children in the family who would have been affected by the Triple P programme if it had been effective. Finally, we reiterate that the overall intensity of intervention was at least as high as that reported in previous non-independent studies claiming positive results [2, 3].

## Assertion 3 the focus on the outcome as childhood mental health difficulties being too narrow

The Triple P programme claims to ‘prevent – as well as treat – behavioral and emotional problems in children’ [Triple P website, accessed 5th March 2018]. This is considered by the developers to occur through changes in parenting practices. Sanders et al. point out that our paper only reports behavioural and emotional problems within the school context, as reported by teachers, rather than the home context, as reported by parents. As the original paper explains, a multi-informant approach would have been desirable but no resources were available for this.

Parental reports of children’s mental health are strongly influenced by the parent’s own state of mind [24, 25], and parental reports of depression, anxiety and stress over the course of a group Triple P intervention generally show improvements in mental state [10].

Given the overwhelming balance of evidence that independent observers fail to report any impact of Triple P on child behaviour [26], and that parental (usually maternal) mood is improved by group attendance, the most parsimonious explanation is that Group Triple P does not have an impact on child behaviour; it simply enables attending parents to think that their child is behaving better. This is clearly a desirable outcome but it is difficult to see why any service commissioner would consider investing in an expensive programme with such limited impact. Further independent research would be valuable in this area.

## Assertion 4 selective reporting of outcomes

Sanders et al., criticise our paper for not presenting the parent reported child mental health outcomes collected during the course of the intervention. These data were only available for a relatively small number of families who completed interventions. Aside from the problems expressed in the previous section about parent-reported child mental health outcomes, the purpose of our paper was to assess whether there had been any effect on *population-level* child mental health difficulties, which the population level Triple P programme purports to produce. These impacts were not found over six years of collected data.

Sanders et al. are correct in that, of the 44.1% of parents who completed a group Triple P intervention once they had enrolled in the programme, mean overall child mental health difficulties were reported by this relatively small number of parents to fall to a modest extent from 15.8 to 12 (on a scale of 0-40, *n* = 366). Parents who completed a group Triple P intervention, however, had children with lower levels of difficulties at the start of the intervention, were more affluent and better educated than those who failed to complete it, suggesting that the parents with children most in need of the intervention would not be receiving it in full [9]. Given the low numbers and completion levels of the more intensive strands of Triple P which do show parental-reported positive impacts in Glasgow city, we would not expect to see an overall impact on population level child mental health difficulties, even if we had surveyed the population of parents in Glasgow, as opposed to just teachers. We would welcome the publication of the qualitative element of the evaluation which gives some insight into parental perceptions of the programme.

## Assertion 5 factual errors and misleading statements

Sanders et al. disagree with our statement that ‘Some doubt has been expressed about the effectiveness of Triple P in deprived communities’ [5]. This statement referred to the meta-analysis of Triple P by Thomas and Zimmer-Gembeck (2007), which concluded that ‘Due to the high number of Triple P studies in the meta-analysis with middle or higher SES, it is not certain that findings can be generalized to low income or high risk groups at this time.’ [27]. This complements our own findings that families from lower SES groups were less likely to complete Triple P interventions [9].

Sanders et al. raise concerns about the lack of information about the cost of the Triple P programme in our paper, despite this being raised as a concern. We requested these cost data formally from NHSGGC, in 2015, and were told that this information was not available. An estimate that we consider very conservative, owing to the lack of inclusion of staff costs in programme delivery, was given in The Times at £4 million [28]. We would welcome the publication of comprehensive cost data for Triple P in Glasgow city.

Sanders et al. assert that our paper ‘failed to take into account that during the intervention period in the same catchment area other parenting interventions were also being supported and implemented concurrently’. From our own knowledge, the only other major parenting programme with significant levels of delivery, the Family Nurse Partnership, began a pilot implementation in Glasgow City in April 2012 [29] with the first cohort of mothers and children completing the programme in late 2014, after the evaluation of Triple P concluded and with first-time parents of children much younger than preschool age – so none of these families would have been included in our analysis. There were a few other parenting programmes operating in Glasgow (e.g. Mellow Parenting, the NCH programme and Incredible Years), however these were operating on a very small scale indeed, not at whole population level, and highly unlikely to have affected overall population level mental health.

The response correctly points out that three cross-references have become misaligned in the final version of the bibliography. We apologise for the typographical errors, but our conclusions are not in any way altered by them and we are happy to offer a corrigendum.

Sanders et al. are mistaken in their claim that our paper says that the protocol was registered: we stated that ‘The protocol for this study was published in 2010’ and a link is provided to the published version. As the evaluation was not a randomised trial, there was no mechanism to formally register it at the time.

## Assertion 6 a number of measurement problems

Despite Sanders et al. claim that there are ‘a number of measurement problems’, they list only two linked difficulties, which were discussed in the original paper. The change in use of the Conduct Problems version was less than ideal but was necessary. It was carried out in response to nursery staff who were finding the 4-16 year old version inappropriate for some preschool children [30]. The alpha measuring internal consistency dropped from .71 to .66, falling just short of the normal level which is seen as acceptable, however one aspect of the creation of this measure is the number of items in the scale, so this fall may simply be a reflection of the change from 5 items to 3 items in the scale – all of which was discussed in the original paper. We believe that the ‘weighting’ which Sanders et al. refer to is the averaging of the 3 items and then multiplying by 5 in order to create a comparable score. In the original documentation and code produced by Youth in Mind, the organisation hosting the SDQ, the averaging and multiplication of 3 items in a normal 5 item measure (where two items within the scale were not completed or incomprehensible, for example) is permitted, so this is no different from usual accepted practice [31]. Sanders et al. state inaccurately that this calculation makes the overall results ‘highly questionable’, however, the conduct problems scale is only one of four scales used to form the Total Difficulties score, and none of the other scales showed improvements over time either.

## Conclusions

The authors strongly reject the contention that our original conclusions were not justified by the evidence. The original paper set out the weaknesses in the study design, however, our methods were robust and would have shown a positive impact of Triple P on the mental health of the population of children in Glasgow city, should there have been one.

We did not agree to perform retrospective subgroup analyses that would show the Triple P interventions in a more favourable light. We consider that independent evaluations should be carried out without the influence of programme developers in order that replicability of results can be truly established.

There is some continuing small scale activity including an independent trial comparing antenatal Triple P with another parenting programme and a highly targeted and structured programme offering level 4 Triple P to a small number of families, but the whole population approach has undoubtedly been abandoned.
